# Dataset for chemorheological and rheokinetic analysis of carbohydrate-HMF-amine adhesives

**DOI:** 10.1016/j.dib.2021.107465

**Published:** 2021-10-09

**Authors:** Catherine Thoma, Pia Solt, Wilfried Sailer-Kronlachner, Thomas Rosenau, Antje Potthast, Johannes Konnerth, Alessandro Pellis, Hendrikus W.G. van Herwijnen

**Affiliations:** a Wood K plus – Competence Center of Wood Composites and Wood Chemistry, Kompetenzzentrum Holz GmbH, Altenberger Str.69, Linz A-4040, Austria; b Institute of Chemistry of Renewable Resources, BOKU-University of Natural Resources and Life Sciences-Vienna, Muthgasse 18, Vienna A-1190, Austria; c Department of Material Science and Process Engineering, Institute of Wood Technology and Renewable Materials, BOKU-University of Natural Resources and Life Sciences-Vienna, Konrad-Lorenz Str. 24, Tulln A-3430, Austria; d Department of Agrobiotechnology IFA-Tulln, Institute of Environmental Biotechnology, BOKU-University of Natural Resources and Life Sciences-Vienna, Konrad-Lorenz Str. 20, Tulln A-3430, Austria

**Keywords:** Hydroxymethylfurfural, Curing, Rheology, Rheokinetics carbohydrates, Adhesives

## Abstract

The work consists of primary and analysed data from rheological measurements of carbohydrate-hydroxymethylfurfural-amine adhesives. The studied adhesives are a bio-based alternative to conventional wood adhesives. The rheological properties were studied at different temperatures in isothermal (80, 90, 95 °C) and non-isothermal (20–120 °C) oscillatory measurements. Non-isothermal rheological measurements were used for the determination of the activation energy based on Vyazovskin's isoconversional method. The viscosity profile of the adhesives, determined from isothermal measurements, was fitted by an empirical model. The viscosity kinetic constant can be obtained from this empirical model and used in further rheokinetic analysis.

Data from density and swelling experiments was measured for the characterization of the adhesive network. The determined polymer-solvent interaction parameter is included in the collected data.

The provided datasets were used in the investigation of the reactivity and curing reaction of the studied adhesives. A discussion and interpretation of the data can be found in the previous publication [1].

## Specifications Table

**Table:** 

Subject	[Materials Science]
Specific subject area	Adhesive characterization, rheological data, rheokinetics, multiwave experiments,
Type of data	Table Figure
How the data were acquired	Data was collected from experimental measurements. For the rheological measurements a rheometer MCR 302 (Anton Paar GmbH) was used with a temperature control unit P-PTD200 (Anton Paar GmbH, Graz, Austria). The RheoCompass 1.22 (Anton Paar, Austria) Software automatically calculated the storage, loss modulus and loss factor of isothermal and non-isothermal measurements. The temperature and time were measured as well. The analysis of the primary data obtained in isothermal, rheological measurements was done using the software ORIGINPro 2016G. This software was used for the fitting of the viscosity profile and the statistical analysis. The density evaluation and mass change of the cured adhesives in the swelling tests is based on experimental values.
Data format	Raw (Primary data) Analyzed
Parameters for data collection	The following variations of the adhesive composition were studied: 2 different amines: Hexamethylenediamine, Bishexamethylenetriamine, different amounts of Hydroxymethylfurfural (HMF) in the adhesives: 0%, 5% or 50%; Multiwave experiments: The experimental data was obtained from rheological measurements. The material response was tested at three different temperatures, 85 °C, 90 °C and 90 °C and at three different frequencies of 10rad/s, 20rad/s and 30rad/s. Isothermal rheological measurements: The data is based on experiments. The material response was tested at three different temperatures of 85 °C, 90 °C and 95 °C. The fitting of the viscosity profile was done up to the sol-gel transition point. Non-isothermal rheological measurements: The data obtained from rheological measurements. The heating rate of the non-isothermal experiments was varied from 1.0 °C/min, 1.5 °C/min, 2 °C/min.
Description of data collection	Primary data was collected from experimental work. In rheological measurements, the data was automatically calculated by the RheoCompass 1.22 (Anton Paar, Austria) Software. An Excel macro [2] was used in the rheokinetic analysis of the rheological data (non-isothermal measurements). Three different pycnometers (each with 10ml) were used for the determination of the adhesive density.
Data accessibility	With the article All data is included as .csv and .tiff files.
Related research article	Thoma, C.; Solt-Rindler, P.; Sailer-Kronlachner, W.; Rosenau, T.; Potthast, A.; Konnerth, J.; Pellis, A.; van Herwijnen, H. W. G., Carbohydrate-hydroxymethylfurfural-amine adhesives: Chemorheological analysis and rheokinetic study. *Polymer* 2021, *231*, 124128. https://doi.org/10.1016/j.polymer.2021.124128. [1]

## Value of the Data

• This dataset is based on the collection of rheological properties at different reaction temperatures and can enable insight into the increased reactivity of the curing reaction of carbohydrate-amine adhesives due to the addition of Hydroxymethylfurfural.
• A remaining challenge in the development of sustainable wood adhesives is the slow curing reaction of the developed adhesives. The dataset can be used by the scientific as well as industrial R&D community as a starting point for further development of sustainable adhesives with a rapid curing behaviour.
• The dataset can be used to assess the potential of bio-based carbohydrate-HMF-amine adhesives. It can be used as comparison to other adhesive systems and enables further data mining to extract insights and uncover patters to guide and accelerate the development of alternative adhesives.
• The activation energies with R^2^ values are calculated using Vyazovskin's model. Data on activation energies that are based on rheological measurements are rarely available in literature.

## 1 Data Description

### 1.1 Analysed data

#### 1.1.1 Multiwave experiments

Table 1A_1 shows the data used for the determination of the sol-gel transition point (time) for all three measured frequencies (10rad/s, 20rad/s, 30rad/s). Multiwave experiments are a standard rheological test method, which allows the measurement of a material sample that is subjected to multiple oscillation frequencies simultaneously. The provided data in this table (Table 1A_1) is based on the primary data of the isothermal, rheological multiwave experiments. (Supporting Information). These multiwave experiments showed that the sol-gel transition point of carbohydrate-amine adhesives occurs at the crossover of storage and loss modulus (tan delta=1).

**Table 1A_1:** Gel point determination: gel point determination for all frequencies as G‘=G‘‘ crossover.

Adhesive	Temperature [°C]	t_G‘=G‘‘_ [s] 10rad/s	t_G‘=G‘‘_ [s] 20rad/s	t_G‘=G‘‘_ [s] 30rad/s	Mean [s]	Standard Deviation [s]
Fru-BHT	85	609	606	606	607	1
	90	417	420	428	421	5
	95	306	310	306	307	2
Fru-HMF(5%)-BHT	85	646	648	652	649	3
	90	469	467	473	470	3
	95	366	356	364	362	4
Fru-HMF(50%)-BHT	85	695	698	702	698	3
	90	522	521	526	523	2
	95	382	380	389	384	4
Fru-HMDA	85	712	722	727	721	6
	90	471	474	495	472	1
	95	422	426	427	425	2
Fru-HMF(5%)-HMDA	85	467	467	457	464	5
	90	401	415	387	394	7
	95	225	236	246	230	6
Fru-HMF(50%)-HMDA	85	711	724	735	723	10
	90	459	461	486	461	1
	95	332	334	371	333	1

Analysed data such as the calculated mean and the standard deviation is included in Table 1A_1 as well. The mean value and standard deviation is calculated based on the time of the sol-gel point at three different frequencies for all carbohydrate-hydroxymethylfrufrual (HMF)-amine adhesives. The low standard deviation supports the assumption that the sol-gel transition point occurs at the crossover of storage and loss modulus.

Fig. 1A_1 depicts the multiwave experiments of hexamethylenediamine (HMDA)-based adhesives such as fructose-HMDA (A–C), fructose-HMF(5%)-HMDA (E-G) and fructose-HMF(50%)-HMDA (I-K) as well as the corresponding standard deviation of the loss factor tan delta measured at three different frequencies for each adhesive (D, H, L). The corresponding information about bishexamethylenetriamine (BHT)-containing adhesives is given in Fig. 1A_2. Both figures contain the primary data of the rheological multiwave experiments.

**Fig. 1A_1:**
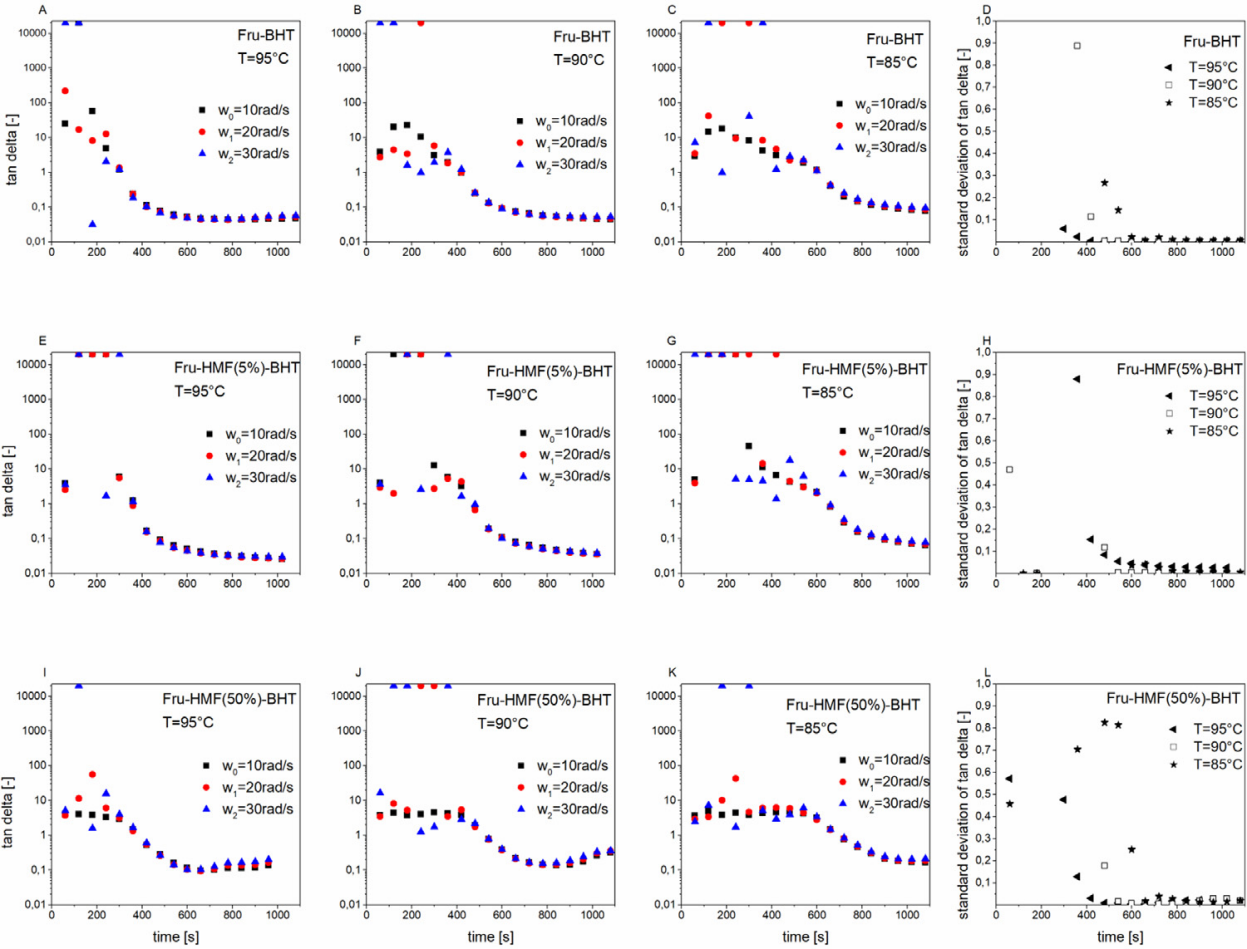
Multiwave experiments of fructose-BHT (A–C), fructose-HMF(5%)-BHT (E–G) and fructose-HMF(50%)-BHT (I–K) as well as the corresponding standard deviation of the loss factor tan delta measured at three different frequencies for each adhesive (D, H, L)

**Fig. 1A_2:**
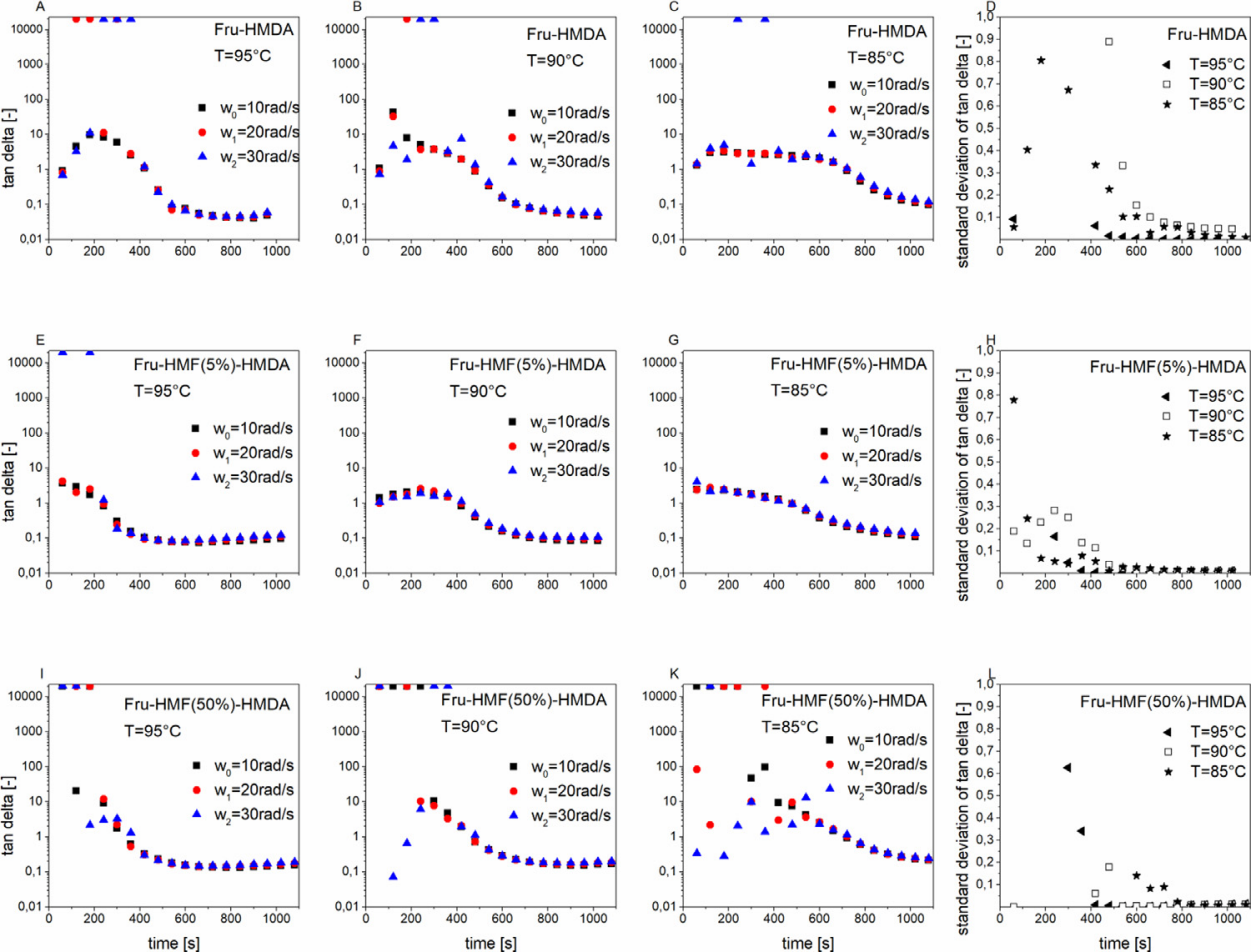
Multiwave experiments of fructose-HMDA (A–C), fructose-HMF(5%)-HMDA (E–G) and fructose-HMF(50%)-HMDA (I–K) as well as the corresponding standard deviation of the loss factor tan delta measured at three different frequencies for each adhesive (D, H, L)

Fig. 1A_1 and Fig. 1A_2 show that the material response becomes frequency independent after the sol-gel transition point is reached (tan delta=1). The obtained values of the sol-gel transition point were used for the gel point determination in Table 1A_1.

#### 1.1.2 Swelling experiments

The crosslink density is an important material characteristic. For the calculation of the crosslink density, it is necessary to evaluate the polymer-solvent interaction parameter. This parameter was determined by swelling experiments at various temperatures. The results of this analysis are given in Table 2A_1, which summarizes the findings on the polymer-solvent interaction parameter for each adhesive and the corresponding R^2^. The linear fit used for the calculation of the polymer-solvent interaction parameter for HMDA-based adhesives is shown in Fig. 2A_1. This calculation was also done for BHT-containing adhesives (Fig. 2A_2). Both figures depict analysed data. The equations used in the calculation are given in the Methods section and the primary data used in the calculation is provided in the Supporting Information.

**Table 2A_1:** Polymer-solvent interaction parameter determined by swelling experiments at various temperatures.

Adhesive	Polymer-Solvent-Interaction Parameter χ_e_ [-]	R^2^ [-]
Fructose-HMDA	0.664	0.969
Fructose-HMF(5%)-HMDA	0.193	0.957
Fructose-HMF(50%)-HMDA	0.912	0.920
Fructose-BHT	0.732	0.498
Fructose-HMF(5%)-BHT	0.787	0.928
Fructose-HMF(50%)-BHT	0.725	0.738

**Fig. 2A_1:**
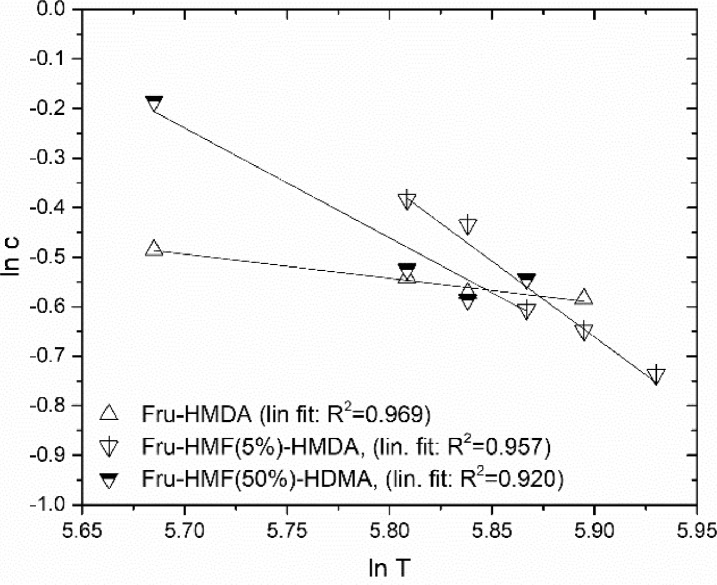
Equilibrium swelling polymer concentration c as function of swelling temperature for HMDA-adhesive samples cured at 120 °C.

**Fig. 2A_2:**
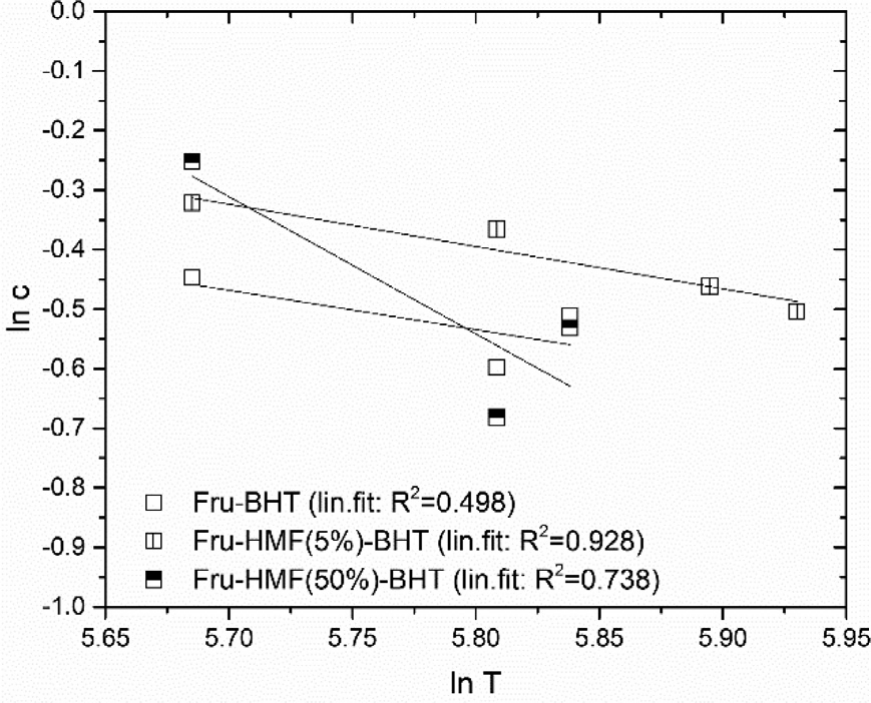
Equilibrium swelling polymer concentration c as function of swelling temperature for BHT-adhesive samples cured at 120 °C.

#### 1.1.3 Data for rheokinetic analysis

The viscosity profile of the curing adhesives obtained in isothermal, rheological measurements was fitted by a linear or exponential fit according to Eqs. (5) and (6). These rheological measurements can be used in the calculation of the activation energy from Arrhenius-type plots. The viscosity kinetic constant K is plotted in logarithmic scale as function of the inverse temperature in an Arrhenius-type plot. The viscosity kinetic constant K can be obtained from the linear or exponential fit Eqs. (5) and ((6)) and is included in the Table 3A_1 together with the corresponding R^2^ for all adhesives.

**Table 3A_1:** Characteristic rheological parameters of fructose-based amine adhesives at isothermal cure temperatures, based on linear or exponential fit of the experimental viscosity data.

			Exponentional Fit		Linear Fit	
Adhesive	T_iso_ [°C]	t_gel_ [s]	K [s^−1^]	R^2^ [-]	K [s^−1^]	R^2^ [-]
Fru-HMDA	85	721	0.0066	0.996	0.0058	0.992
	90	472	0.0118	0.989	0.0102	0.978
	95	425	0.0254	0.990	0.0197	0.914
Fru-BHT	85	607	0.0087	0.996	0.0078	0.990
	90	421	0.0133	0.992	0.0124	0.984
	95	307	0.0361	0.999	0.030	0.951
Fru-HMF (5%)-HMDA	85	464	0.0873	0.999	0.0083	0.992
	90	401	0.0146	0.998	0.0139	0.994
	95	230	0.0317	0.999	0.0299	0.997
Fru-HMF (5%)-BHT	85	649	0.0076	0.994	0.0065	0.986
	90	470	0.0094	0.988	0.0084	0.978
	95	362	0.0155	0.996	0.0144	0.989
Fru-HMF (50%)-HMDA	85	723	0.0108	0.99988	0.0010	0.992
	90	461	0.0171	0.999	0.0137	0.986
	95	333	0.0299	0.999	0.0244	0.985
Fru-HMF (50%)-BHT	85	698	0.0074	0.990	0.0064	0.982
	90	523	0.0110	0.985	0.0095	0.971
	95	384	0.0130	0.990	0.0120	0.980

The multiwave experiments Fig. 1A_1 and Fig.1A_2) showed that after the gel point the material response of the studied adhesives is frequency-independent. The calculation of the conversion using ((7) is valid from the gel point onward. Before the gel point, the moduli have frequency-dependent material response, consequently the influence of the frequency on the calculated conversion (7) was determined (see Table 4A_1). It was found that the standard deviation (SD) of α is very small, SD(α) <1 %. This makes the calculation of the extent of conversion based on the loss modulus (7) valid. Table 4A_1_gives the degree of conversions α calculated based on the loss modulus G’’ (Eq. (7)) of fructose-BHT measured at isothermal conditions at different frequencies ω= 10rad/s, 20rad/s and 30rad/s. Table 4A_1 contains analysed data.

**Table 4A_1:** Degree of conversions α calculated based on the loss modulus G’’ (Eq. (8)) of fructose-BHT measured at isothermal conditions (85 °C) at different frequencies ω= 10rad/s, 20rad/s and 30rad/s.

Time [s]	α [%] Based on G‘‘ (FruBHT) ω=10rad/s	α [%] Based on G‘‘ (FruBHT) ω=20rad/s	α [%] Based on G‘‘ (FruBHT) ω=30rad/s	Standard Deviation (SD) alpha [%]
120	0.02	*-0.28*	*-0.47*	0.2
180	0.05	0.00	*-0.77*	0.4
240	0.13	*-0.05*	0.49	0.2
300	0.27	0.31	0.30	0.0
360	0.51	0.29	0.30	0.1
420	0.90	0.73	0.71	0.1
480	1.64	1.79	2.56	0.4
540	3.74	3.98	3.78	0.1
600	6.09	5.50	5.10	0.4
660	7.50	6.97	6.82	0.3
720	11.21	9.68	9.17	0.9
780	14.45	13.18	11.88	1.1
840	18.39	15.95	15.19	1.4
900	22.86	20.24	18.86	1.7
960	26.96	24.17	22.80	1.7
1020	31.41	28.54	26.81	1.9
1080	35.62	32.82	31.21	1.8
1140	40.20	37.33	35.54	1.9
1200	43.97	41.58	40.27	1.5
1260	48.85	46.26	44.62	1.7
1320	53.11	50.77	49.45	1.5
1380	57.58	55.40	54.00	1.5
1440	61.83	60.05	59.01	1.2
1500	66.70	65.00	63.89	1.2
1560	70.95	69.63	68.85	0.9
1620	75.88	74.90	73.91	0.8
1680	80.59	79.49	78.87	0.7
1740	85.53	84.74	83.94	0.6
1800	90.50	90.01	89.59	0.4
1860	95.13	95.09	94.50	0.3
1920	100.00	100.00	100.00	0.0

Table 4A_2 gives the activation energy as function of conversion calculated based on Vyazovskin's method from non-isothermal temperature sweeps.

**Table 4A_2:** Data for calculation of effective activation energy of fructose-BHT adhesive and fructose-HMF(5%)-BHT adhesive.

Fructose-BHT					Fructose-HMF(5%)-BHT				
	Temperature [K]					Temperature [K]			

Conversion [1]	1K/min	1.5K/min	2K/min	E_A [kJ/mol]	Conversion [1]	1K/min	1.5K/min	2K/min	E_A [kJ/mol]
0,01	358,17	361,19	366,2	94,5	0,01	356,18	362,19	364,2	65,6
0,02	361,17	363,19	372,2	69,8	0,02	357,18	365,18	365,2	41,6
0,04	363,17	364,19	376,19	57,6	0,04	359,18	370,19	371,2	33,8
0,06	364,17	366,19	378,19	54,4	0,09	362,18	374,19	375,2	29,6
0,09	366,17	368,18	379,19	59,4	0,12	363,14	375,19	377,19	31,4
0,12	367,15	369,19	380,19	60,7	0,16	364,18	377,18	178,2	27,8
0,14	368,17	370,19	381,19	60,8	0,2	365,18	378,18	380,19	28,4
0,16	369,17	371,19	382,2	60,7	0,25	366,18	379,18	381,19	28,4
0,2	370,17	372,19	383,18	60,7	0,3	367,22	380,18	382,19	29,6
0,25	375,17	376,19	386,19	73,6	0,41	369,18	382,18	384,19	29,6
0,3	374,17	375,19	385,19	72,4	0,47	370,18	383,19	385,19	29,6
0,34	375,17	376,19	386,19	73,6	0,53	371,18	384,18	386,19	29,6
0,45	379,17	379,18	387,19	98,4	0,63	373,18	386,18	387,19	28,8
0,53	381,17	381,18	388,19	116,6	0,72	375,17	388,18	389,19	28,4
0,63	384,17	384,17	389,19	165,4	0,82	376,18	390,18	390,19	25,6
0,72	386,17	386,18	390,19	210,1	0,91	378,17	391,18	392,18	29,6
0,91	391,17	397,18	392,18	880					

### 1.2 Primary data

The following additional data of the chemorheological and rheokinetic analysis is included in the Supporting information.

Table 1A_2, Table 1A_3 and Table 1A_4 (provided in Supporting Information) contain the primary data of the rheological multiwave experiments of BHT-containing adhesives. The primary data of the multiwave test of HMDA-containing adhesives is include in Table 1A_5, 1A_6 and 1A_7 of the Supporting Information.

Primary as well as analysed data from the swelling experiments of adhesives are included in Table 2A_2 (Supporting Information). The primary data was collected for the calculation of the polymer-solvent interaction parameter and contains the initial adhesive mass, the adhesive density, solvent density as well as the adhesive mass at equilibrium after swelling. Analysed data, needed for the calculation, is included as well. The primary data used in the determination of the adhesive density is given in Table 2A_3 (Supporting Information). This table also contains the calculated mean values of the adhesive density and the standard deviation. A pycnometer was used for the density measurements. In preliminary experiments, the volume of the pycnometer was measured and the density of tetrahydrofuran (THF) was determined as control. For completeness, the results of this preliminary work is given in Table 2A_4 (Supporting Information).

The viscosity profile of the curing adhesive was determined in isothermal, rheological measurements. An exponential as well as a linear fit were used to describe the experimental values. The primary data of isothermal rheological measurements performed at 85 °C, 90 °C and 95 °C are given in Table 3A_ 6 (Supporting Information). The primary data (complex viscosity and time in logarithmic scale) obtained from isothermal rheological measurements was fitted with a linear regression, data of this linear fit is listed in Table 3A_2 (Supporting Information). The parameters of the linear fit, such as intersection with y-axis and slope, together with statistical analysis of the linear fit are given in Table 3A_3 (Supporting Information). Data obtained from the exponential fit of the isothermal rheological measurements are in Table 3A_4 (Supporting Information) and the statistical analysis and parameters of the fit can be found in Table 3A_5.

Primary data of non-isothermal, oscillatory rheological measurements is included in Table 4A_3 of the Supporting Information. This dataset contains primary data of fructose-BHT and fructose-HMF(5%)-BHT adhesives. These fits were used for the determination of the viscosity kinetic constant (Table 4A_1).

## 2 Experimental Design, Materials and Methods

### 2.1 Materials

The analysed fructose-Hydroxymethylfurfural-amine adhesive were developed as an alternative to conventional fossil-based wood adhesives [1]. The reactivity of adhesives, especially the cure speed and temperature-dependent material behaviour are essential criterions in the development of new adhesives [3].

Six different adhesives were studied: Fructose-Bishexamethylenetriamine (Fru-BHT), Fructose-Hydroxymethylfurfural (5%)-Bishexamethylenetriamine (Fru-HMF5%-BHT), Fructose-Hydroxymethylfurfural (50%)-Bishexamethylenetriamine (Fru-HMF(50%)-BHT), Fructose-Hexamethylenediamine (Fru-HMDA), Fructose-Hydroxymethylfurfural(5%)-Hexamethylenedia-mine (Fru-HMF(5%)-HMDA, Fructose-Hydroxymethylfurfural(50%)-Hexamethylenediamine (Fru-HMF(50%)-HMDA). The adhesives varied in the type of amine used, either BHT or HMDA as well as the amount of Hydroxymethylfurfural, 5% or 50% based on fructose content. The molar ratio was 3.9:1=fructose:BHT for BHT based adhesives and 2.6:1 for fructose-HMDA adhesives.

### 2.2 Multiwave experiments

Rheological measurements were done using the rheometer MCR 302 (Anton Paar GmbH). The measurements were done under ambient air (relative humidity 50%) using a temperature control unit P-PTD200 (Anton Paar GmbH, Graz, Austria). The disposable, parallel plate diameter was 25.0 mm and the corresponding gap size was 1 mm.

Multiwave experiments allows the use of multiple frequencies in one dynamic oscillatory test. Three different frequencies (10rad/s, 20rad/s, 30rad/s) were applied in the measurement of each adhesive sample. The dynamic oscillatory measurements were performed at three different temperatures of 85 °C, 90 °C or 95 °C. The samples were heated to the set temperature within 20 s, then the temperature was kept constant. A fundamental frequency of ω_0_=10rad/s and an initial strain of 2% was used for the multiwave experiments, both being well within the linear viscoelastic regime (LVE) of all tested adhesives. The other applied frequencies (harmonics) were ω_1_=20rad/s and ω_2_=30rad/s with an amplitude factor of 0.5. The resulting maximum amplitude was 3.14%, which was well within the LVE region. The loss factor tan (delta) is independent of the applied frequency in the gel point. The tested adhesives had a frequency independent response also after the gel point (see Fig. 1A_1 and Fig. 1A_2). The gel point was found at tan=1 (see Table 1A_1).

### 2.3 Density measurements

The prepolymer adhesive samples were cured at 120 °C for 2 h in an oven at atmospheric pressure. The density was measured using three 10ml pycnometer and an analytical balance (Sartorius CPA225, *d* = 0.01 mg (100 g)). The density of the adhesives was measured at least three times at 21.7 °C, each with a different pycnometer. The volume of the pycnometers was determined with water. This reproducibility of the density measurements was checked by determining the density of Tetrahydrofuran (THF). The cured adhesive samples are a porous material, therefore they were crushed with a pestile to obtain a homogeneous powder. This powder was then used in the density measurements, 2-propanol was used as solvent. The mean value of the density was determined and used for further calculations.

### 2.4 Swelling experiments

The adhesive prepolymers were cured at 120 °C for 2 h in an oven at atmospheric pressure. Approximately 20 mg of a cured adhesive sample were used in the measurements and put together with 2 ml DMSO in closed vials. These closed vials were then put in an oven at different temperatures for 24 h, then the solvent was removed and the adhesive samples were weighted again. In cases in which the adhesives didn't withstand the treatment at higher temperatures, the data was excluded from the analysis. At least three measurements at different temperatures were used for the calculations.

The polymer-solvent interaction parameter was calculated from the measured density and mass change after swelling experiments (see Table 2A_1). The primary data (initial adhesive mass $m_{0}$, polymer density$\;\rho_{P}$, adhesive mass at equilibrium swelling$\;m_{\infty }$) was used for the calculation of crosslink density $\nu_{E\_swell}\;$and molecular weight per elastically effective network chain $M_{C}$ to (1) and (2)

(1) $$\nu_{E\_swell}=\frac{M_{c}}{\rho_{P}}$$

(2) $$M_{C}=\frac{-V_{S}\rho_{P}\left(c^{\frac{1}{3}}-\frac{c}{2}\right)}{\ln\left(1-c\right)+c+\chi c^{2}}$$

With V_s_ being the molar volume of the solvent and the concentration c (3).

(3) $$c=\frac{m_{0}}{\rho_{P}V_{\infty }}$$

(4) $$V_{\infty }=\frac{m_{0}}{\rho_{P}}+\left(\frac{m_{\infty }-m_{0}}{\rho_{S}}\right)$$

$\rho_{S}$ is the density of the solvent.

The polymer-solvent interaction parameter can be calculated based on the temperature dependence of the polymer concentration at equilibrium swelling c. (see Fig. 2A_1 and Fig. 2A_2).

### 2.5 Isothermal rheological measurements

Isothermal rheological were done with the rheometer MCR 302 (Anton Paar GmbH) under ambient air (relative humidity 50%) using a temperature control unit P-PTD200 (Anton Paar GmbH, Graz, Austria). A disposable, parallel plate diameter of 25.0 mm and a corresponding gap size of 1 mm was used.

The fitting of the viscosity profile is based on primary data from isothermal rheological measurements at three different temperatures 80 °C, 90 °C and 95 °C. The isothermal measurements were performed until the adhesives were fully cured. For the further analysis only the data before the sol-gel transition point could be used. The sol-gel transition point was determined at tan=1, which is based on multiwave experiments. Only the data of the viscosity profile (viscosityη as function of time t) up to the sol-gel transition point was used for the analysis. The viscosity of the adhesive samples up to the sol-gel transition point was fitted by an exponential function (5), where $\eta_{0}$ is the zero shear viscosity and *K* is the viscosity kinetic constant.

(5) $$\eta =\eta_{0}*e^{Kt}$$

In addition, a linear fit (6) from the data in logarithmic scale was done as well.

(6) $$\ln\left(\eta \right)=\ln\left(\eta_{0}\right)+Kt$$

### 2.6 Non-isothermal rheological measurements

Primary data for the rheokinetic analysis using Vyazovskin's isoconversional method [4] was obtained from non-isothermal oscillatory measurements. The data was obtained with the rheometer MCR 302 (Anton Paar GmbH) using a temperature control unit P-PTD200 (Anton Paar GmbH, Graz, Austria). The disposable, parallel plate diameter was 25.0 mm and the corresponding gap size was 1 mm.

Non-isothermal dynamic measurements are also referred to as temperature sweeps. The temperature-sweeps were done from 20 °C to 120 °C with an angular frequency of 10rad/s and an initial strain of 1%. The heating rates were 1.0 °C/min, 1.5 °C/min and 2.0 °C/min.

The conversion $\alpha_{t}$ calculated based on the loss modulus, which is normalized between 0 and 1 according to (7).

(7) $$\alpha_{t}=\frac{G_{t}-G_{ti}}{G_{tf}-G_{ti}}$$

### 2.7 Vyazovskin's isoconversional method

For the rheokinetic analysis, Vyazovskin's isoconversional method [4] was applied to the data from non-isothermal measurements. There are two basic assumptions of isoconversional methods [5]. The first one is that the rate of a process is a function of temperature and conversion (8) and that (8) is the product of the two functions, which are independent of each other.

(8) $$\frac{d\alpha }{dt}=\phi \left(T,\alpha \right)$$

The rate of progress can be formally described as

(9) $$\frac{d\alpha }{dt}=k\left(T\right)*f\left(\alpha \right)$$

k(T) is considered the rate and is only dependent on temperature and f(α) is the conversion function, which is only dependent on the conversion [5,6]. Replacing k(T) with the Arrhenius equation leads to the following Eq. (10):

(10) $$\frac{d\alpha }{dt}=A*\exp\left(-\frac{E}{RT}\right)*f\left(\alpha \right)$$

The second assumption is that the activation parameters can be obtained from a set of runs, such as temperature versus heating rate. Vyazovkin's method [4] is based on Eq. (11), in which the apparent activation energy (E_A_) at a specific conversion α can be determined by finding the minimum of the equation $\phi (E_{A}$) for a series of experiments (*n*) performed with different heating rates (β_i, j_).

(11) $$\phi \left(E_{A}\right)=\;\sum_{i=1}^{n}\sum_{j\neq i}^{n}\frac{I\;\left(E_{A,\alpha },\;T_{\alpha ,i}\right)\beta_{j}}{I\;\left(E_{A,\alpha },\;T_{\alpha ,j}\right)\beta_{i}}$$

The function $I\;(E_{A,\alpha },\;T_{\alpha ,i})$ is defined as:

(121) $$I\;\left(E_{A,\alpha },\;T_{\alpha ,i}\right)=\;\int_{0}^{T}\exp\left(-\frac{E}{RT}\right)dT$$

Where the values of I can be obtained by numerical integration. An Excel macro published by Joseph et al. [2] was used for the calculations of the activation energy based on Vyazovskin's model [4].

Table 4A_2 give the calculated effective activation energy for fructose-BHT and fructose-HMF(5%)-BHT based on Vyazovskin's method.

## CRediT authorship contribution statement

**Catherine Thoma:** Conceptualization, Methodology, Formal analysis, Writing – original draft. **Pia Solt:** Conceptualization, Writing – review & editing. **Wilfried Sailer-Kronlachner:** Writing – review & editing. **Thomas Rosenau:** Supervision, Writing – review & editing. **Antje Potthast:** Supervision, Writing – review & editing. **Johannes Konnerth:** Supervision, Resources, Writing – review & editing. **Alessandro Pellis:** Resources, Data curation. **Hendrikus W.G. van Herwijnen:** Conceptualization, Resources, Writing – review & editing, Funding acquisition.

## Declaration of Competing Interest

The authors declare that they have no known competing financial interests or personal relationships which have or could be perceived to have influenced the work reported in this article.

## Appendix Supplementary materials

Image, application 1
